# Soybean Cytochrome b5 Is a Restriction Factor for Soybean Mosaic Virus

**DOI:** 10.3390/v11060546

**Published:** 2019-06-11

**Authors:** Hexiang Luan, Haopeng Niu, Jinyan Luo, Haijian Zhi

**Affiliations:** National Center for Soybean Improvement, Nanjing Agricultural University, Nanjing 210095, China; luanhexiang87@163.com (H.L.); niuhaopeng@126.com (H.N.); 2015101173@njau.edu.cn (J.L.)

**Keywords:** P3 protein, cytochrome b5, yeast two-hybrid, virus-induced gene silencing

## Abstract

Soybean mosaic virus (SMV) is one of the most destructive viral diseases in soybeans (Glycine max). In this study, an interaction between the SMV P3 protein and cytochrome b5 was detected by yeast two-hybrid assay, and bimolecular fluorescence complementation assay showed that the interaction took place at the cell periphery. Further, the interaction was confirmed by co-immunoprecipitation analysis. Quantitative real-time polymerase chain reaction analysis revealed that *GmCYB5* gene was differentially expressed in resistant and susceptible soybean plants after inoculation with SMV-SC15 strain. To test the involvement of this gene in SMV resistance, the *GmCYB5* was silenced using a bean pod mottle virus (BPMV)-based vector construct. Results showed that *GmCYB5-1* was 83% and 99% downregulated in susceptible (NN1138-2) and resistant (RN-9) cultivars, respectively, compared to the empty vector-treated plants. Silencing of *GmCYB5* gene promotes SMV replication in soybean plants. Our results suggest that during SMV infection, the host CYB5 protein targets P3 protein to inhibit its proliferation. Taken together, these results suggest that CYB5 is an important factor in SMV infection and replication in soybeans, which could help soybean breeders develop SMV resistant soybean cultivars.

## 1. Introduction

Soybean (*Glycine max* (L.) Merr.) is an important protein and oil crop. Soybean mosaic virus (SMV), a member of *Potyvirus*, is seed-borne and aphid-transmitted. SMV causes severe reduction of soybean yield and destroys the seed quality [1,2]. In the United States, 98 isolates of SMV were divided into seven strains, namely G1 to G7, according to differential phenotypic reactions to five resistant and two susceptible varieties of soybeans [3,4]. Resistance loci containing dominant resistance (R) genes named *Rsv1*, *Rsv3*, and *Rsv4* were mapped to chromosomes 13, 14, and 2, respectively [5,6,7,8,9]. *Rsv1* was resistant to G1–G6 [10] while *Rsv3* was resistant to strains G5–G7 [5]. *Rsv4* was initially thought to provide resistance against all North American strains of SMV but later was shown to exhibit a late susceptible phenotype to strains G1 and G2 [11,12,13,14,15]. Based on the reaction to specific soybean cultivars, the SMV isolates were classified into 21 strains in China and were named SC1 to SC21 [16,17,18]. Resistance derived from the resistant to SC (*Rsc*) loci to the strains from China have been mapped to chromosome 2 in Kefeng 1, chromosome 13 in Qihuang and chromosome 14 in Dabaima, respectively [19,20,21].

SMV has a single-stranded positive sense RNA genome of ~9.6 kb in length, which is similar to other *Potyvirus*, in processing to eleven mature multi-functional proteins. Of these, the P3 protein is reported to participate in viral replication, movement, virulence, pathogenesis and in one case avirulence [22,23,24,25,26,27,28,29,30,31]. However, the precise biochemical function of SMV P3 protein, and the potyviral P3 protein in general, is largely uncharacterized due to the lack of structural or functional motifs in its sequence. Recent researches revealed that the P3 cistron plays a crucial role in SMV virulence on Rsv1 and Rsv4 soybean genotypes [32,33]. The P3 protein has also been reported to interact with ribulose-1,5-bisphosphate carboxylase/oxygenase (RuBisCO), thus affecting the systematic development of host plants [34]. The endoplasmic reticulum (ER)-localized SMV P3 targets eukaryotic elongation factor 1 alpha (eEF1A) to facilitate its survival in the soybean plant [35]. SMV P3 was also used to generate RNAi-mediated silencing plants. Transgenic lines exhibited stable and enhanced resistance to other *Potyvirus* [36].

Cytochrome B5 (CYB5) is a class of heme proteins associated with endoplasmic reticulum in plants, animals, and fungi. As a ubiquitous intercellular electron transporter, CYB5 participates in various redox reactions in cells thereby regulating the balance of reactive oxygen species (ROS) in plants. In plants, amino acid sequences of CYB5 have been identified in cauliflower [37], tobacco [38], and rice [39]. Sequence analysis showed that these proteins shared common characteristics of carboxyl terminal polar parts rich in positively-charged amino acids [40]. Previous studies have focused on the structural aspects of the CYB5 interaction with CYP450 monooxygenases [41] and the biochemical and kinetic aspects of CYB5 involved in the CYP450 monooxygenase reaction [42]. However, the function of CYB5 enzymes in virus infections, especially *Potyvirus* infections are unknown.

Based on the SMV P3 interaction network, a protein named GmCYB5 encoded by the Glyma18G154900 gene was selected for further characterization. Here, we studied the role of GmCYB5 in the process of SMV infection. We showed that GmCYB5 inhibited SMV proliferation by targeting the virus protein P3.

## 2. Materials and Methods

### 2.1. Plant Growth and Virus Strains

Soybean (*Glycine max* (L.)) cultivars NN1138-2 and RN-9, which are susceptible and resistant to SMV SC15 strain, respectively, were grown in an aphid-free greenhouse with day and night temperatures of 25 °C and 20 °C, in 65% relative humidity and during a 14 h photoperiod. We used SMV-SC15 strain in this study, which is one of the most virulent strains in China [18]. Fully expanded unifoliate leaves were mechanically inoculated by SMV-SC15. NMY51 strain of yeast was used in yeast two-hybrid analysis (Dualsystems Biotech, Schlieren, Switzerland), which is an ideal reporter strain for DUAL membrane screening systems which can be used to find novel interaction partners of a protein of interest by screening cDNA libraries, and compatible with most LexA based yeast two-hybrid systems. All the materials were provided by the National Center for Soybean Improvement, Nanjing Agricultural University, China.

### 2.2. Yeast Two-Hybrid Assay

A soybean cDNA library (~0.68 × 10^7^ clones) from SMV-SC15 infected soybean (cv. NN1138-2) was cloned into the modified vector pPR3-N using Gateway technology. The P3 gene of SMV-SC15 was cloned in pBT3 and used as a bait to screen the library (3× clones) by co-transformation in yeast (NMY51). Yeast transformants expressing P3-interacting proteins were selected on synthetic dropout medium lacking tryptophan (Trp), leucine (Leu), histidine (His), and adenine (Ade). Yeast strains expressing P3 interactors were further assessed for *β*–galactosidase activity.

### 2.3. Interaction of GmCYB5-1 and P3 Using Y2H

The full length of GmCYB5-1 was cloned into prey vector pPR3 by the gateway system. The NMY51 yeast cells harboring the pBT3-P3 reporter plasmid was transformed with pPR3-GmCYB5-1 plasmid. The transformants were placed on agar plates with synthetic media containing dextrose lacking Leu, Trp, His, and Ade (SD^-Leu-Trp-His-Ade^), and incubated for two days at 30 °C. The surviving transformants were re-cultured until OD_600_ 0.8 and dropped at concentrations ranging from 1:10, 1:100, 1:1000 to 1:10,000 on SD^-Leu-Trp-His-Ade^ medium with α-x-gal at final concentration of 4 mg/mL to determine the interaction affinity.

### 2.4. Bimolecular Fluorescence Complementation (BiFC) Assays

BiFC assays were carried out as described before [43]. The interaction proteins were cloned to pSITE-n/cEYFP vectors [44] and transformed into *Agrobacterium tumefaciens* strain LBA4404. Positive agrobacteria which fused with reciprocal halves of EYFP were co-infiltrated into transgenic *N. benthamiana* plants expressing nuclear localized H2B protein with a CFP tag [45]. Leaf tissues were immersed in water after 2 days and checked by confocal microscopy using PLAPO60XWLSM (NA 1.0) objective. The interaction was confirmed using both combinations of reciprocal nEYFP/cEYFP fusion proteins in two separate experiments (three replicates per experiment).

### 2.5. Sequence Analysis of GmCYB5

To amplify *GmCYB5-1*, primers were designed by Primer Premier5.0 software (Premier Biosoft, Palo Alto, CA, USA) (Supplemental Table S1). Based on the Williams82 soybean reference genome annotation Glyma v1.1, the coding sequence (CDS) region of the *GmCYB5-1* gene was cloned by polymerase chain reaction (PCR) using soybean cDNA as templates. The sequence alignment and phylogenetic analysis were performed by DNASTAR package [46].

### 2.6. Expression Analysis of GmCYB5

When the first pair of true leaves had developed, RN-9 and NN1138-2 plants were rub-inoculated by SMV-SC15. Leaf samples were collected from infected plants at 0, 2, 8, 12, and 24 h post-inoculation. In addition, root, stem, leaf, flower, and pod were sampled from NN1138-2 plants without virus inoculation. All samples were in triplicates and flash frozen in liquid nitrogen immediately after collection and subsequently stored at −80 °C until use. Total RNA was extracted by TRIZOL using a total RNA isolation kit (Tiangen, Beijing, China). cDNA was synthesized using HiScript ® II QRT SuperMix according to the manufacturer’s instructions (Vazyme Biotech, Nanjing, China). The expression levels of target genes were analyzed by qRT-PCR using a Light-Cycler 480 (Roche Diagnostics, Indianapolis, IN, USA). Three technical replicates were performed for each biological replicate. The target gene sequences used in this study were obtained from Soybean Genome Sequences. Primers were designed to amplify gene specific PCR products of <200 bp in size. Primer sequences are listed in Supplemental Table S1. B-Tubulin was used as an internal control to normalize the cDNA concentrations. qRT-PCR was carried out in a 96-well plate using SYBR Green Mix, with cycling program as 95 °C for 1 min followed by 40 cycles of 95 °C for 10 s and 60 °C for 30 s. Gene expression was quantified using the relative quantification (2^−ΔΔCt^) method. Each sample or treatment was tested in at least three biological replicates and the same experiment was performed twice. 

### 2.7. Construction of Recombinant Plasmids, in Vitro Transcription, and Plant Inoculation

The BPMV vectors, pGG7R1 and pGG7R2, were provided by the National Center for Soybean Improvement, Nanjing Agricultural University, China. To construct the VIGS vector, a 126 bp (E3-D44) cDNA fragment conserved in GmCYB5 isoforms was amplified from NN1138-2 with the primers listed in Supplemental Table S1. The PCR product was inserted into the pGG7R2 vector. After transformation into DH5α competent cells, plasmids were extracted and then sequenced by Invitrogen Biotechnology Co. Ltd (Shanghai, China). Resulting sequences were analyzed by BLAST to confirm that the target fragment was inserted into the vector.

FpGG7R2-GmCYB5 construct, carrying the fragment to silence the *GmCYB5,* and pGG7R1 vector were transcribed after linearizing with *Sal* I and *Sal* I/*Not* I, respectively. Briefly, capped RNA transcripts were synthesized by incubating 30 μL of linearized plasmids in 50 μL of reaction mixture containing 10× Buffer, T7 RNA polymerase, rGTP, rATP, rCTP, rUTP, m^7^G^5^’cap, RNase inhibitor, and DEPC H_2_O at 37 °C for 2 h. In vitro transcription products of pGG7R1 and pGG7R2-CYB5 were mixed in a ratio of 1:1 to produce an infectious transcript, and then immediately inoculated on the fully expanded primary leaves of 7-day-old NN 1138-2 to generate the CYB5-silenced plant (S_CYB5_). For silencing *CYB5* gene in RN-9 plant, the desiccated leaf tissues from of NN1138-2-S_CYB5_ which contain the infectious transcript (pGG7R2-CYB5 and pGG7R1) were mixed with inoculation buffer and then rub-inoculated on RN-9 primary leaves. Empty-vector (V) inoculated plants served as mock-inoculated controls.

### 2.8. Silencing Efficiency of GmCYB5

To confirm target gene silencing efficiency, treatment groups were inoculated with S_CYB5_, and control group were inoculated with V. When plants mosaic symptoms showed up, the infected leaves were collected and the RNA expression levels of *GmCYB5* were analyzed by qRT-PCR.

### 2.9. Resistance Analysis of S_CYB5_

When the first pair of trifoliolate leaves developed, resistant (RN-9) and susceptible (NN1138-2) soybean cultivars were inoculated with tissue from CYB5 silencing plants, and V as the control. When the second pair of trifoliolate leaves developed, all the plants were inoculated with SMV-SC15. SMV-SC15-infected leaves from RN-9 and NN1138-2 were collected at 0, 4, 7, 10, and 14 dpi in triplicates, flash frozen in liquid nitrogen immediately, and stored at −80 °C.

The RNA accumulation of SMV was analyzed by qRT-PCR using SMV-CP (coat protein) gene primers (Supplemental Table S1), and SMV-CP protein concentration was analyzed by double-antibody sandwich-ELISA.

## 3. Results

### 3.1. P3 Protein Interact with GmCYB5-1 

**Figure 1:**
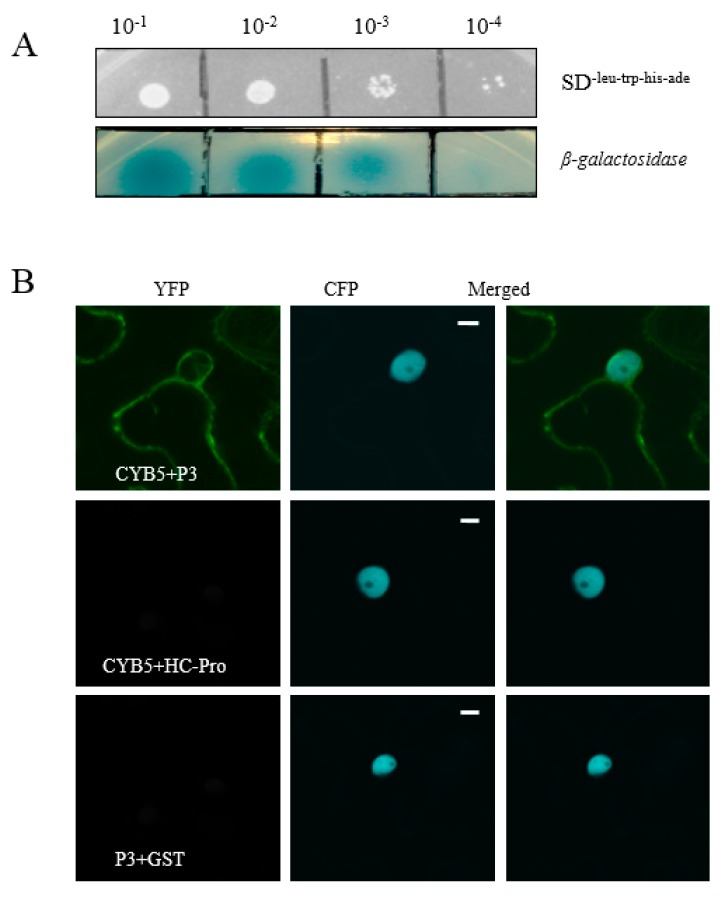
GmCYB5-1 interacts with the P3 protein of soybean mosaic virus (SMV). (**A**) Interaction of SMV-P3 and GmCYB5 in yeast growing on selective medium SD^-leu-trp-his-ade^ with X-α-gal at different concentrations. Images are representative two independent experiments for each interaction. (**B**) Bimolecular florescence complementation (BiFC) micrographs of 40× magnification at 48 h post-infiltration from plants co-expressing nYFP-GmCYB5 and cYFP-P3, with cYFP-HC-Pro and cYFP-GST are negative controls. Transgenic *Nicotiana benthamiana,* expressing CFP-H2B (nuclear localized histone 2B), were used for the BiFC assays. Images are representative of three separate infiltrations from two independent experiments for each interaction using both combinations of n/cYFP fused proteins. Scale bars: 10 µm.

SMV P3 protein is a virulence determining factor. During SMV infection, there might be some host proteins promoting its infection and survival in the host plant. Therefore, we conducted a yeast two-hybrid (Y2H) screen to identify soybean proteins that interacted with the SMV P3 protein. Among the several identified P3-interacting proteins, GmCYB5-1 (Glyma18g154900) was selected for further characterization. The full length GmCYB5-1 was amplified from NN1138-2 and constructed into a yeast vector. The cells which contain both GmCYB5-1 and P3 were grown on synthetic dropout SD^-Leu-Trp-His-Ade^ plates with and without X-α-gal at the dilutions of (1:10, 1:100, 1:1000, and 1:10,000). The results showed strong binding affinity between GmCYB5 and P3 proteins at 1:10 and 1:100 dilutions and weak binding affinity at 1:10,000 dilution (Figure 1A).

Interaction between P3 and GmCYB5-1 proteins was also confirmed by bimolecular florescence complementation (BiFC) assays. In BiFC assays the reciprocal N- or C-terminal halves of enhanced yellow fluorescent proteins (nEYFP and cEYFP) were used to label the proteins, then labeled proteins were co-expressed in tobacco transiently. The results revealed that P3 interacted with GmCYB5-1 in plants; as YFP florescence was detected in plants co-infiltrated with P3 and GmCYB5-1. In contrast, P3 did not interact with GST (glutathione-S-transferase) and GmCYB5-1 did not interact with SMV helper component-proteinase (HC-Pro) (Figure 1B). Together, these results suggested that P3 could interact with GmCYB5-1.

### 3.2. Sequence Analysis of GmCYB5

**Figure 2:**
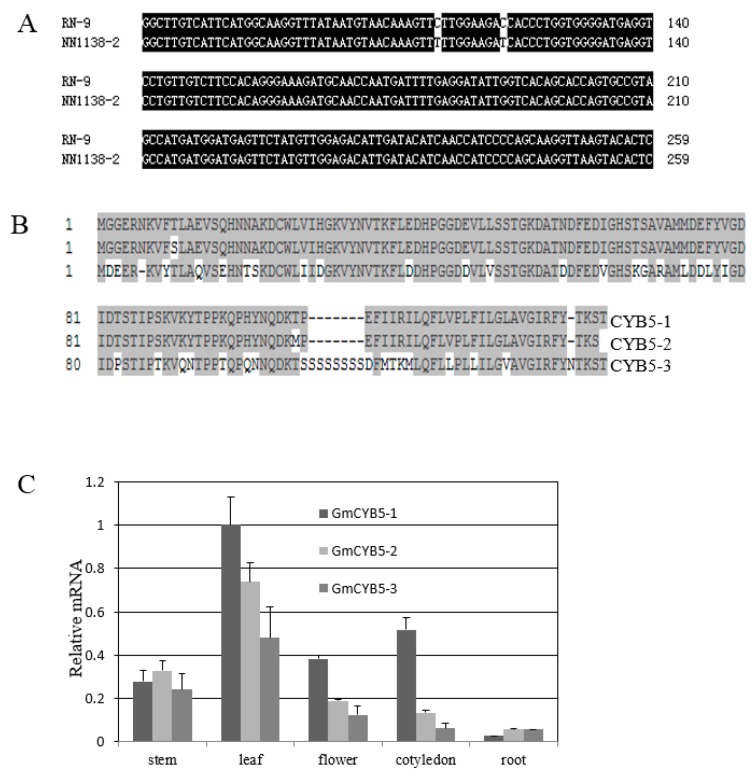
Sequence analysis of GmCYB5. (**A**) The full cDNA sequence alignment of *GmCYB5-1* from resistant (RN-9) and susceptible (NN1138-2) cultivars. (**B**) Amino acid sequence alignment of GmCYB5 isoforms was carried out using ClustalW in the Megalign program of the DNASTAR package, with identical residues shaded in gray. Numbers indicate the position of amino acid residues. Results are representative of two to three independent experiments. (**C**) Relative mRNA levels of *GmCYB5* isoforms in different tissues of soybean plants (cv. NN1138-2) as determined by quantitative RT-PCR analysis. Error bars indicate SD (*n* = 3).

**Figure 3:**
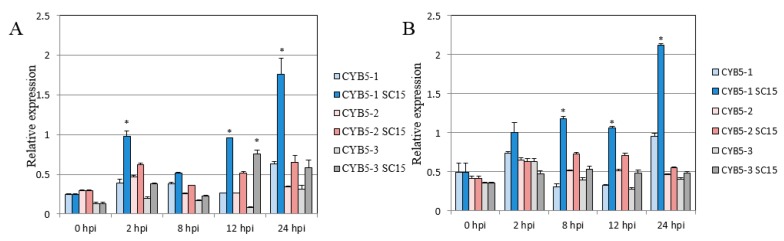
Analysis of mRNA levels of *CYB5* isoforms in soybean. Relative mRNA levels of *GmCYB5* isoforms in (**A**) susceptible plants (cv. NN1138-2) or (**B**) resistant plants (cv. RN-9) with or without SMV (SC15) infection. Samples are collected at 0, 2, 8, 12, and 24 h post infection (hpi) and quantified by qRT-PCR. Error bars indicate SD (*n* = 3). Asterisks denote the significant difference between pathogen-treated and control plants for each respective isoform, *t*-test, *p* < 0.001. Results are representative of two–three independent experiments.

The full length of GmCYB5-1 was amplified and cloned from both susceptible (NN1138-2) and resistant (RN-9) soybean cultivars. By sequence alignment two single nucleotide polymorphisms (SNPs) were identified in GmCYB5-1 of the two cultivars (Figure 2A). The *Glycine. max* genome contains three GmCYB5-like sequences including Glyma18g154900 (GmCYB5-1), Glyma03g080100 (GmCYB5-2), and Glyma08g354100 (GmCYB5-3); their predicted protein sequences shared a minimum of 70% and maximum of 98% identity with one another (Figure 2B). In order to determine the expression levels of *GmCYB5* isoforms in different tissues, a quantitative (q)RT-PCR analysis was carried out and the results showed that transcript levels of *GmCYB5* isoforms were higher in leaf tissue comparing to other analyzed tissues (cotyledon, stem, flower, and roots) (Figure 2C). To determine if GmCYB5 was involved in SMV infection, the expression pattern analysis was carried out in both susceptible (NN1138-2) and resistant (RN-9) cultivars with or without SMV infection using qRT-PCR. The three isoforms of *GmCYB5* were all induced after infection with strain SC15 of SMV at the indicated time point in both susceptible and resistant plants (Figure 3A,B). Furthermore, the expression level of *GmCYB5-1* was much higher than other isoforms after SMV inoculation. Taken together, these results indicated that GmCYB5 might be involved in SMV infection.

### 3.3. Silencing of GmCYB5

**Figure 4:**
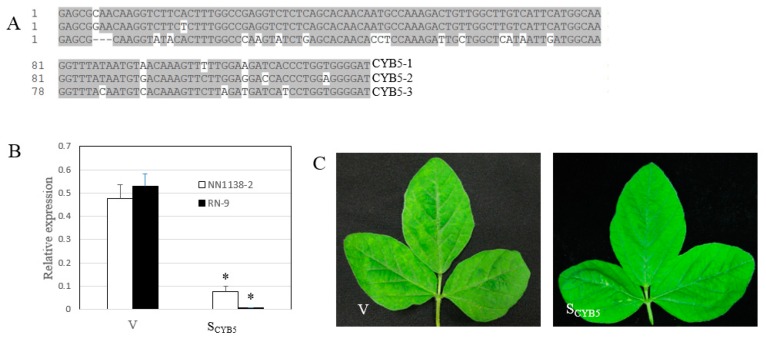
Silencing *GmCYB5* in soybean. (**A**) Sequence of *GmCYB5* isoforms used for silencing. Sequence alignment was carried out using ClustalW in the Megalign program of the DNASTAR package, with identical residues shaded in gray. (**B**) Relative mRNA levels of *GmCYB5-1* isoform in soybean plants (cv. NN1138-2 and RN-9) infected with empty vector (V), or vector targeting *GmCYB5-1* (S_CYB5_) as determined by qRT-PCR analysis. Error bars indicate SD (*n* = 3). Asterisks denote significant difference in expression of the respective transcripts when compared to V plants, *t*-test, *p* < 0.001. Results are representative of two independent experiments. (**C**) Comparing symptoms of NN1138-2 plants with *GmCYB5* mRNA expression (S_CYB5_) knocked down and that infected with the control bean pod mottle virus (BPMV) vector (V).

We next tested the role of GmCYB5 in resistance of soybean to SMV. We knocked down the *GmCYB5* genes in soybean using the bean pod mottle virus (BPMV)-based VIGS (virus-induced gene silencing) vector [47,48]. A 126-nt fragment of GmCYB5-1 from NN1138-2 cultivar which showed high similarity among the three *GmCYB5* isoforms, was selected and cloned (Figure 4A). The cloned fragment was verified by sequencing and subsequently inserted into the modified pGG7R2 vector. The pGG7R2-GmCYB5-1 construct was expected to knock down the expression of all isoforms simultaneously because the various isoforms share >82% nucleotide identity in the 126 nt fragment. Inoculated plants (cv. NN1138-2 and RN-9) with the construct were tested for *GmCYB5-1* transcript level using qRT-PCR. Notably, mRNA level of *GmCYB5-1* was reduced by 83% in the susceptible plant (NN1138-2) and 99% in the resistant plant (RN-9) in the *GmCYB5* knock down plants (S_CYB5_) compared to control plants (inoculated with a BPMV control vector; containing a non-specific sequence, V) (Figure 4B). Silencing of *GmCYB5* did not change the morphology of soybean plants.

### 3.4. Knock Down of GmCYB5 Expression Promote SMV Accumulation in Soybean Plants

**Figure 5:**
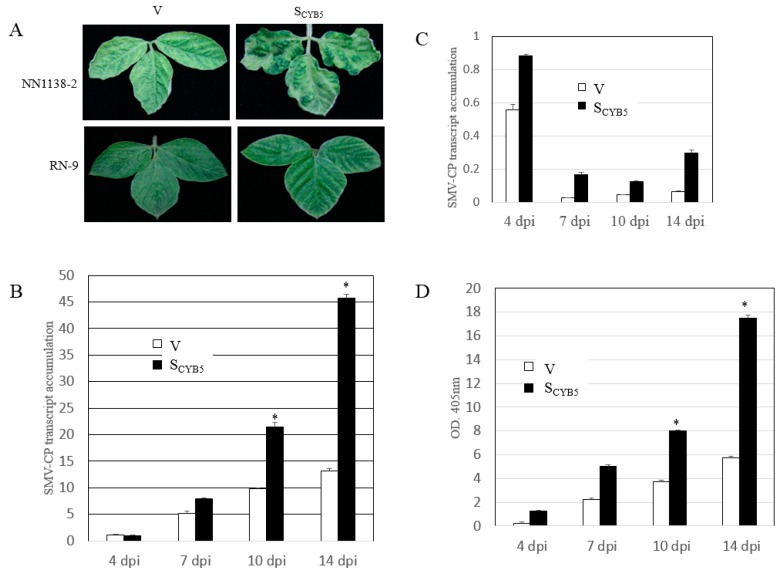
Silencing *GmCYB5* alters soybean response to SMV. (**A**) Visual symptoms of SMV-SC15 on *GmCYB5-*silenced plant (S_CYB5_) (right panels), control plant inoculated with BPMV vector (V) (left panels), and susceptible plant (NN1138-2) (upper panels) and resistant plants (RN-9) (lower panels). (**B**) qRT-PCR analysis of SMV coat protein (CP) level at indicated dpi in V and S_CYB5_ plants of susceptible cultivar (NN1138-2) and (**C**) resistant cultivar (RN-9). Error bars indicate SD (*n* = 3). Asterisks denote significant differences from V for each time point, *t*-test, *p* < 0.001. Results are representative of two to three independent experiments. (**D**) ELISA results of SMV CP level in V and S_CYB5_ plants (cv. NN1138-2) inoculated with SMV-SC15 at indicated dpi (4, 7 dpi from I, 10 and 14 dpi from S) with SMV SC15. Error bars indicate SD (*n* = 3). Asterisks denote significant difference from V, *t*-test, *p* < 0.001. Results are representative of three independent experiments.

The V and S_CYB5_ plants (cvs. NN1138-2 and RN-9) were infected with the SC15 strain (virulent on NN1138-2, avirulent on RN-9) of SMV. The NN1128-2-S_CYB5_ plants developed more severe SMV-related symptoms compared to V plants (Figure 5A upper panel). In contrast, there was no visible SMV symptom of S_CYB5_ and V on RN-9 plants (Figure 5A lower panel). This correlated with the enzyme-linked immunosorbent assay (ELISA) analysis results of total protein extracts, which showed that there was no SMV accumulation in either V or S_CYB5_ RN-9 plants. Furthermore, SMV infection resulted in much higher induction of SMV coat protein (CP) expression in the S_CYB5_ plants than in V plants of NN1138-2 (Figure 5B). The expression level of SMV CP transcript was measured in RN-9 plants (S_CYB5_ and V) using qRT-PCR. SMV CP mRNA was detected at a low level at 4 dpi and increased in response to SMV infection in the S_CYB5_ plants (Figure 5C). The CP protein of SMV was detected by ELISA in S_CYB5_ and V plants of NN1138-2 cultivar. Based on the ELISA result there was more CP in S_CYB5_ plants than V plants in both inoculated (I) and systemic (S) leaves (Figure 5D). Together, these results indicated that knock down of *GmCYB5* expression reduced the plant resistance to SMV.

## 4. Discussion

Protein–protein interactions are fundamental to the development and survival of viruses in host plants and can serve as excellent targets for identifying interacting factors related to host-pathogen interactions. A widely used method to characterize the function of known proteins is to identify their interacting factors and infer their roles based on the functions of known interacting factors. Here, we identified a protein of soybean plant (CYB5-1) that interacted with SMV P3 protein using yeast two-hybrid screening. However, as this screening uses DUAL membrane system which can result in a certain number of false positives [49], BiFC and co-immunoprecipitation were used to confirm the reliability of interaction. In the BiFC assay, the fluorescence was detected at cell periplasm. Characterizing soybean gene function by gene transformation has limited value due to low efficiency and high cost. However, the BPMV-VIGS system offers a convenient way to downregulate gene expression and was successfully introduced to analyze the candidate gene function in soybean [50]. In this research, the CYB5 function during SMV infection was characterized by BPMV-VIGS system. Since three isoforms of *GmCYB5* share high sequence similarity, a conserved region was selected to generate the BPMV-VIGS construct to silence the genes to the maximum extent possible. By qRT-PCR, a 83% reduction of *GmCYB5* transcript accumulation in the susceptible cultivar (NN 1138-2) and 99% reduction in the resistant cultivar (RN-9) were observed, as compared with the vector control plants (inoculated with an empty vector). The VIGS systems do not completely silence the expression of target genes. The silencing efficiency can be affected by inserted regions [51], cultivars [52], and environment conditions even for the same plant [53]. 

We next investigated whether silencing of *GmCYB5* gene altered the soybean resistance to SMV. Severe SMV symptoms were observed on the S_CYB5_ plants compared to V plants. These results were consistent with mRNA accumulation of SMV CP that was measured by qRT-PCR in susceptible cultivar (NN1138-2) and resistant cultivar (RN-9), as well as the virion concentration that is represented by the level of SMV CP in susceptible cultivar (NN1138-2) in the ELISA result.

Previous research showed that the difference in resistance to SMV SC15 between RN-9 and NN1138-2 was mainly attributed to the *GmPEX14* gene [54]. In this study, sequence analysis showed that there are two single nucleotide polymorphisms (SNPs) in *GmCYB5-1* coding region between RN-9 and NN1138-2. After SMV inoculation, the resistant RN-9 plants showed extreme resistance, and no SMV symptom at all. However, a small amount of SMV was detected by qRT-PCR in inoculated leaves. A possible biochemical mechanism of extreme resistance could be the direct antiviral effect of reactive oxygen species (ROS) accumulation at infection sites [55]. The resistant RN-9 plants have accumulated a high level of ROS early after SMV inoculation [54]. The accumulation of ROS is usually linked to the host plant defense responses during the plant–virus interactions. A slower host response allows a certain degree of virus replication and movement resulting in oxidative stress including the ROS and programmed death of affected plant cells before conferring pathogen arrest (hypersensitive response, HR) [56]. 

CYB5 is known to be an electron transfer component in a number of oxidative reactions in biological tissues. Since CYB5 has relatively high mid-point redox potential, it can deliver only the second electron to oxyferrous CYP450 [57]. In fact, most studies suggest that with certain P450 forms and certain substrates, CYB5 supplies electrons to the intermediate oxygenated-ferrous-P450-substrate complex more rapidly than does NADPH-P450 oxidoreductase, which is accompanied by a decrease in H_2_O_2_ formation by the enzyme [58]. The symptom development associated with compatible cucumber mosaic virus- and zucchini yellow mosaic virus-infected plants showed an enhanced lipid peroxidation indicating an advanced disintegration of membranes [59]. The increased H_2_O_2_ accumulation in sunflower prevented an early increase in antioxidant activities after the Sunflower chlorotic mottle virus (SuCMoV) infection which thus can facilitate virus systemization in compatible interactions [60]. The strong systemic burst of H_2_O_2_ in Plum pox virus (PPV)-infected plants that exhibited severe symptoms might be attributed, at least in part, to a delayed and failed attempt by the host to elicit resistance to the virus in systemic tissues [61,62,63]. This agrees with other findings that demonstrate the pivotal role of timely ROS accumulation in virus resistance [64,65]. Similarly, as GmCYB5 was silenced in the susceptible cultivar, the host plants showed more susceptibility to SMV. This observation suggests that GmCYB5 is required for cellular ROS accumulation which is important in resistance to SMV by host plants.

## Supplementary Materials

The following are available online at https://www.mdpi.com/1999-4915/11/6/546/s1, Table S1: Primer sequence.
