# Supplementary material for: Soybean Cytochrome b5 Is a Restriction Factor for Soybean Mosaic Virus
Source: Viruses. 2019 Jun 11;11(6):546. doi: 10.3390/v11060546 (PMC6631803; doi:10.3390/v11060546)
Supplement: Supplementary file 1 [file viruses-11-00546-s001.pdf]

Supplementary materials

Table 1. Primer sequence.

**Gene name**	**Primer sequence （5′→3′）**
GmCYB5-1 F	AGCTGGCCAATGGGTGGGGAGCGCAAC
GmCYB5-1 R	CACTGGCCATGTTGATTTGGTGTAGA
qCYB5-1F	TCTCAGCACAACAATGCCAA
qCYB5-1R	GTTGCATCTTTCCCTGTGGA
SilCYB5-1F	TAAGGATCCGAGCGCAACAAGGTCTTCAC
SilCYB5-1R	TTATGGCCAATCCCCACCAGGGTGATCTTC
SMV-CP F	TTCTGAAAGTCCGTATATGCCTAG
SMV-CP R	GCCTTTCAGTATTTTCGGAGTT
Tubulin F	GGAGTTCACAGAGGCA GAG
Tubulin R	CACTTACGCATCACATAGCA
